# Mitogen-activated protein kinase 6 negatively regulates secondary wall biosynthesis by modulating MYB46 protein stability in *Arabidopsis thaliana*

**DOI:** 10.1371/journal.pgen.1009510

**Published:** 2021-04-07

**Authors:** Jong Hee Im, Jae-Heung Ko, Won-Chan Kim, Brent Crain, Daniel Keathley, Kyung-Hwan Han

**Affiliations:** 1 Department of Horticulture, Michigan State University, East Lansing, Michigan, United States of America; 2 National Academy of Agricultural Science, Rural Development Administration, Jeonju, Republic of Korea; 3 Department of Plant & Environmental New Resources, College of Life Science and Graduate School of Biotechnology, Kyung Hee University, Yongin-si, Gyeonggi-do, Republic of Korea; 4 School of Applied Biosciences, Kyungpook National University, Daegu, Republic of Korea; 5 Department of Forestry, Michigan State University, East Lansing, Michigan, United States of America; The University of North Carolina at Chapel Hill, UNITED STATES

## Abstract

The R2R3-MYB transcription factor MYB46 functions as a master switch for secondary cell wall biosynthesis, ensuring the exquisite expression of the secondary wall biosynthetic genes in the tissues where secondary walls are critical for growth and development. At the same time, suppression of its function is needed when/where formation of secondary walls is not desirable. Little is known about how this opposing control of secondary cell wall formation is achieved. We used both transient and transgenic expression of MYB46 and mitogen-activated protein kinase 6 (MPK6) to investigate the molecular mechanism of the post-translational regulation of MYB46. We show that MYB46 is phosphorylated by MPK6, leading to site specific phosphorylation-dependent degradation of MYB46 by the ubiquitin-mediated proteasome pathway. In addition, the MPK6-mediated MYB46 phosphorylation was found to regulate *in planta* secondary wall forming function of MYB46. Furthermore, we provide experimental evidences that MYB83, a paralog of MYB46, is not regulated by MPK6. The coupling of MPK signaling to MYB46 function provides insights into the tissue- and/or condition-specific activity of MYB46 for secondary wall biosynthesis.

## Author summary

Secondary cell walls are critical for plant growth and of economic importance to humans as fiber, pulp for paper, and as a renewable source of energy. A master switch for secondary wall biosynthesis, MYB46, ensures the exquisite expression of the biosynthetic genes in the tissues where secondary walls are critical. However, suppression of secondary wall formation is needed during impermanent cessation of vegetative growth triggered by environmental stresses. Although the expression of *MYB46* is upregulated by abiotic stresses, secondary wall formation may not occur. How is this opposing control achieved? The mitogen-activated protein kinase (MPK) cascade is among the most conserved signal transduction systems in eukaryotes and plays a crucial role in the regulation of biochemical and physiological changes associated with environmental stimuli and phytohormones. In this report, we show that MYB46 is negatively regulated by MPK6 during salt stress, providing a novel insight into a mechanism by which plants incorporate environmental signals into differential regulation of secondary wall biosynthesis.

## Introduction

Secondary cell walls, located between the plasma membrane and primary cell wall, are a defining feature of xylem fibers and vessels, providing mechanical support for plants and serving as a conduit for long-distance transport of water and solutes. They constitute the majority of plant biomass and are of economic importance to humans as fiber, animal feed, pulp for manufacture of paper, and as an environmentally desirable, cost-effective, renewable source of energy. The biosynthesis of secondary walls occurs in a highly-coordinated manner by successive deposition of cellulose fibrils, hemicelluloses and lignin upon cessation of cell growth [1–3]. This process requires a coordinated transcriptional activation of the biosynthetic genes for the components, suggesting the existence of one or more central transcriptional regulators.

The plant-specific R2R3-MYB transcription factor MYB46 is a master switch for the secondary wall biosynthetic program in *Arabidopsis thaliana*, and ensures the exquisite expression of the secondary wall biosynthetic genes in the secondary wall forming tissues [3–8]. Proper function of MYB46 is critical for secondary wall formation, but the coordinated interplay of this signal with environmental stimuli poses interesting questions, particularly when cells are exposed to biotic or abiotic stresses that induce the expression of the *MYB46* gene [9] while suppression of secondary wall formation is necessary in those affected tissues. For example, upon exposure to abiotic stresses, growth ceases earlier in the aerial parts than in the root system, which allows the stressed plants to reallocate resources to root growth [10], requiring concomitant suppression of MYB46 activity in the aerial portions along with induction of the response in the root system. How does the plant that is undergoing stress-induced upregulation of *MYB46* transcription manage to suppress MYB46 function in cells where secondary wall formation is not needed? How does the plant incorporate extracellular (e.g., environmental, positional) signals into the condition-specific activity of MYB46? Little is known about the mechanism regulating this condition-specific activity of MYB46.

The mitogen-activated protein kinase (MPK) cascade is among the most conserved signal transduction systems in eukaryotes and plays a crucial role in the regulation of biochemical and physiological changes associated with environmental stimuli and phytohormones. Specific MPKs are activated in response to extracellular stimuli. *Arabidopsis* MPK6 is activated by abiotic stresses, such as salt, cold, wounding and hyper-osmotic stresses [11, 12]. In *Arabidopsis*, there are approximately 570 MPK phosphorylation substrates. Transcription factors, involved in the regulation of development, defense, and stress responses, represent the largest group of MPK substrates [13]. While the importance of the MPK signal cascade in plant growth and development is well-known, no single MPK-mediated regulation has been described in secondary wall biosynthesis.

In this report, we present that MYB46 is post-translationally regulated by MPK6, which might function as an efficient means to manage acute responses to salt stress.

## Results

### MPK6 phosphorylates MYB46 protein with a direct interaction

**Fig 1:**
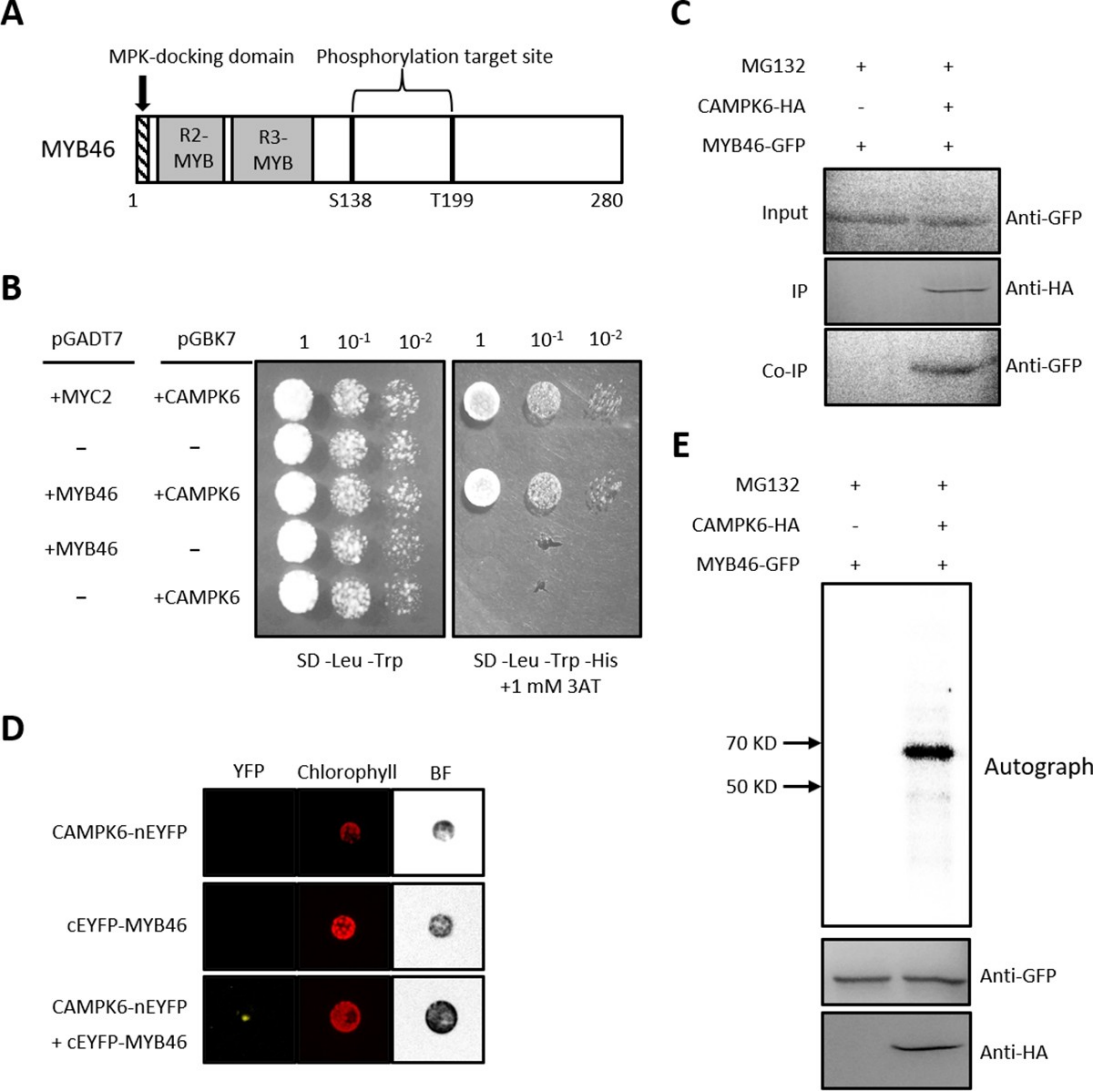
MPK6 phosphorylates MYB46 protein with a direct interaction. (A) Schematic drawing of MPK docking domain and phosphorylation target sites in MYB46 predicted by Eukaryotic Linear Motif (http://elm.eu.org/). (B) Yeast two hybrid assay showing interaction between MYB46 and CAMPK6 proteins. A standard spot assay was carried out using the designated selective media condition (-Leu, -Trp, -His in the presence of 1 mM 3AT). (C) Co-immunoprecipition of MYB46 and CAMPK6. GFP conjugated MYB46 (MYB46-GFP) and HA conjugated CAMPK6 (CAMPK6-HA) were co-expressed in AMPs with MG132 treatment. After expression, immunoprecipitation was carried out with anti-HA antibody and then protein blot analysis was carried out with anti-GFP antibody. (D) Bimolecular fluorescence complementation (BiFC) of MYB46 and CAMPK6. BiFC was carried out with designated combinations in AMPs with MG132 treatment. (E) Immunocomplex kinase assay of MYB46 and CAMPK6. MYB46-GFP and CAMPK6-HA were expressed in AMPs. After the expression, immunoprecipitation was carried out with anti-HA antibody and anti-GFP antibody, then *in vitro* kinase assay was carried out.

Eukaryotic Linear Motif (ELM; http://elm.eu.org) prediction tool showed that MYB46 contains a putative mitogen-activated protein kinase (MPK)-docking domain (^2^RKPEVAI^8^) near the N-terminal and two potential phosphorylation target sites (S138 and T199) (Fig 1A). In MPK signaling, docking domains act as substrate determinants, recruiting kinases to the correct substrates and thereby enhancing their fidelity and efficiency of action [14]. In addition to the docking domains, phosphorylation target sites (i.e., phosphoacceptor motifs) on substrates contribute to MPK specificity. This result suggests that MYB46 may be subject to post-translational regulation by MPK.

To test this hypothesis, we first investigated whether MYB46 interacts with MPKs known to be associated with salt stress responses. We used MPK3 and 6, which has been shown to be activated by salt stress [15, 16]. Since MPK3 did not regulate MYB46 transcriptional activity and protein stability (S1 Fig), we focused on MPK6. Yeast 2-hybrid (Y2H) experiments using MYB46 as bait and a constitutively active form of MPK6 (i.e., MPK6 ^D218G, E222A^; CAMPK6, hereafter) as a prey showed that MYB46 interacts with CAMPK6 (Fig 1B). In this Y2H experiment, the interaction of MYC2 and CAMPK6 was included as a positive control (Fig 1B) [17]. To confirm the Y2H results, we performed a co-immunoprecipitation assay using the *Arabidopsis* mesophyll protoplast transient expression system (AMPs)[18]. Both *MYB46-GFP* and *CAMPK6-HA* fusion constructs were transiently co-expressed in AMPs in the presence of the proteasome inhibitor MG132. CAMPK6 interacting protein was immunoprecipitated using anti-HA antibody followed by protein blot analysis with anti-GFP antibody. The result suggested a direct interaction of these two proteins (Fig 1C). In addition, we carried out BiFC experiments in AMPs. As shown in Fig 1D, CAMPK6 interacted with MYB46.

We then investigated whether the interaction of MYB46 and CAMPK6 resulted in MYB46 phosphorylation. Immunocomplex kinase assay with CAMPK6 showed that CAMPK6 phosphorylates MYB46 protein (Fig 1E).

### MYB46 protein stability is negatively regulated by CAMPK6

**Fig 2:**
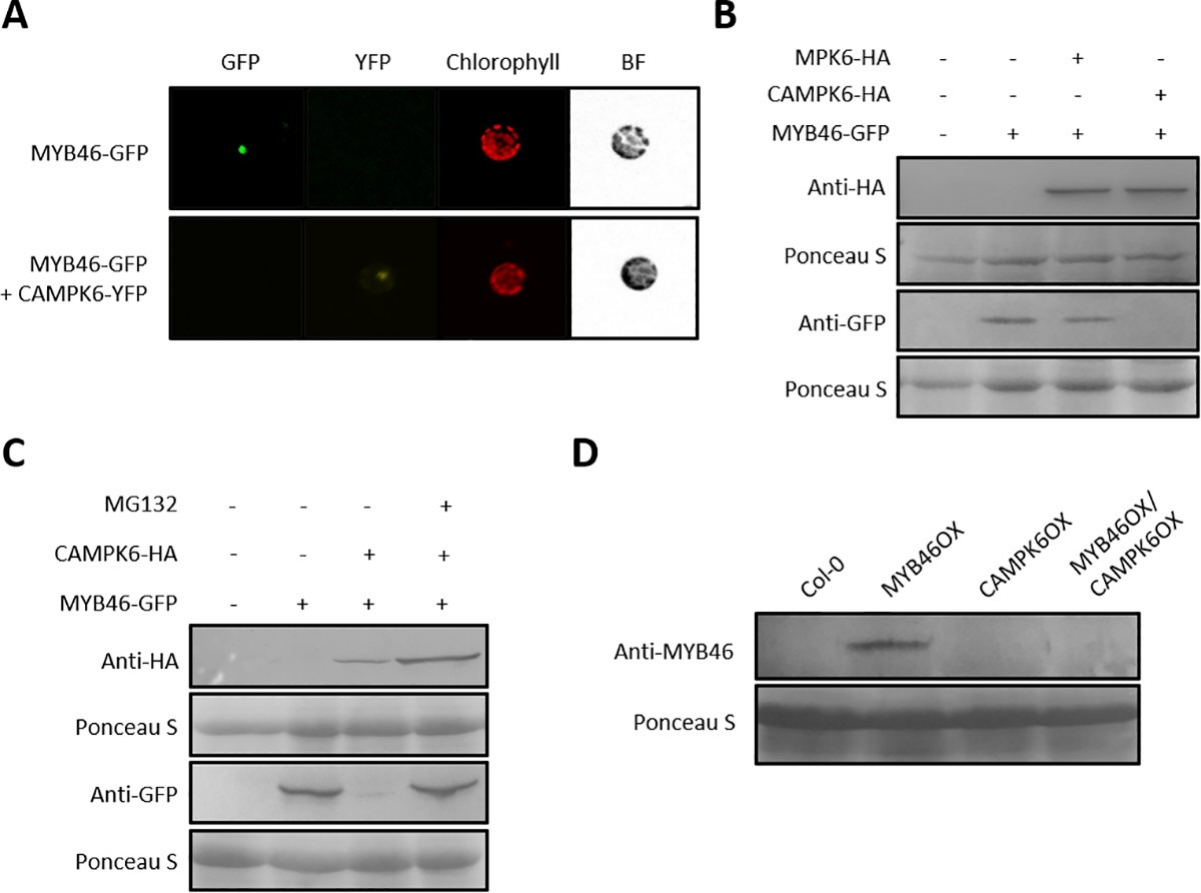
MYB46 protein stability is negatively regulated by CAMPK6. (A) *MYB46*-*GFP* fusion construct was expressed in AMPs with or without *CAMPK6*-*YFP*. Image was taken after incubation for 10-hr by fluorescence microscopy. (B) Protein blot analysis of MYB46. MYB46-GFP was expressed in AMPs with or without MPK6-HA or CAMPK6-HA for 10-hr. After expression, the cells were harvested for protein blot analysis with anti-HA antibody or anti-GFP antibody. (C) Protein blot analysis of MYB46 with or without MG132. MYB46-GFP was expressed in AMPs with or without CAMPK6-HA. For MG132 treatment, 1 ul of 5 mM of MG132 was added to 1 ml reaction and incubated for an additional 9-hr. The harvested cells were used for protein blot analysis with anti-GFP antibody or anti-HA antibody. (D) Protein blot analysis of MYB46. Using total protein extracted from transgenic *Arabidopsis* plants overexpressing MYB46 (MYB46OX), CAMPK6 (CAMPK6OX), or both (MYB46OX/CAMPK6OX), MYB46 protein was detected with anti-MYB46 antibody.

To examine the stability of MYB46 protein in the presence of CAMPK6, we expressed *MYB46-GFP* fusion construct (*35S*::*MYB46*-*GFP*) in AMPs with or without *CAMPK6-YFP* fusion construct (*35S*::*CAMPK6*-*YFP*). GFP signal was detected when *MYB46-GFP* was expressed alone, but the signal disappeared with C*AMPK6-YFP* co-expression (Fig 2A), suggesting that MYB46 might be degraded when co-expressed with CAMPK6. This observation was confirmed by protein blot analysis of MYB46-GFP fusion proteins expressed in AMPs with or without CAMPK6-HA. MYB46 protein level was not much changed with co-expression of MPK6, a wild-type MPK6, but disappeared in the presence of CAMPK6 (Fig 2B). However, MYB46 protein degradation was not observed with addition of proteasome inhibitor MG132 (Fig 2C), suggesting that CAMPK6 may degrade MYB46 protein through the ubiquitin-mediated proteasome pathway [19]. This CAMPK6-mediated degradation of MYB46 was further confirmed *in planta* using transgenic *Arabidopsis* plants overexpressing *MYB46* with *CAMPK6* together (i.e., MYB46OX/CAMPK6OX) or only *MYB46* (i.e., MYB46OX) or only *CAMPK6* (i.e., CAMPK6OX) (Fig 2D). Protein blot analysis using anti-MYB46 antibody showed that the level of MYB46 protein was decreased in the MYB46OX/CAMPK6OX plants (Fig 2D).

### MPK6 negatively regulates MYB46 function *in planta*

**Fig 3:**
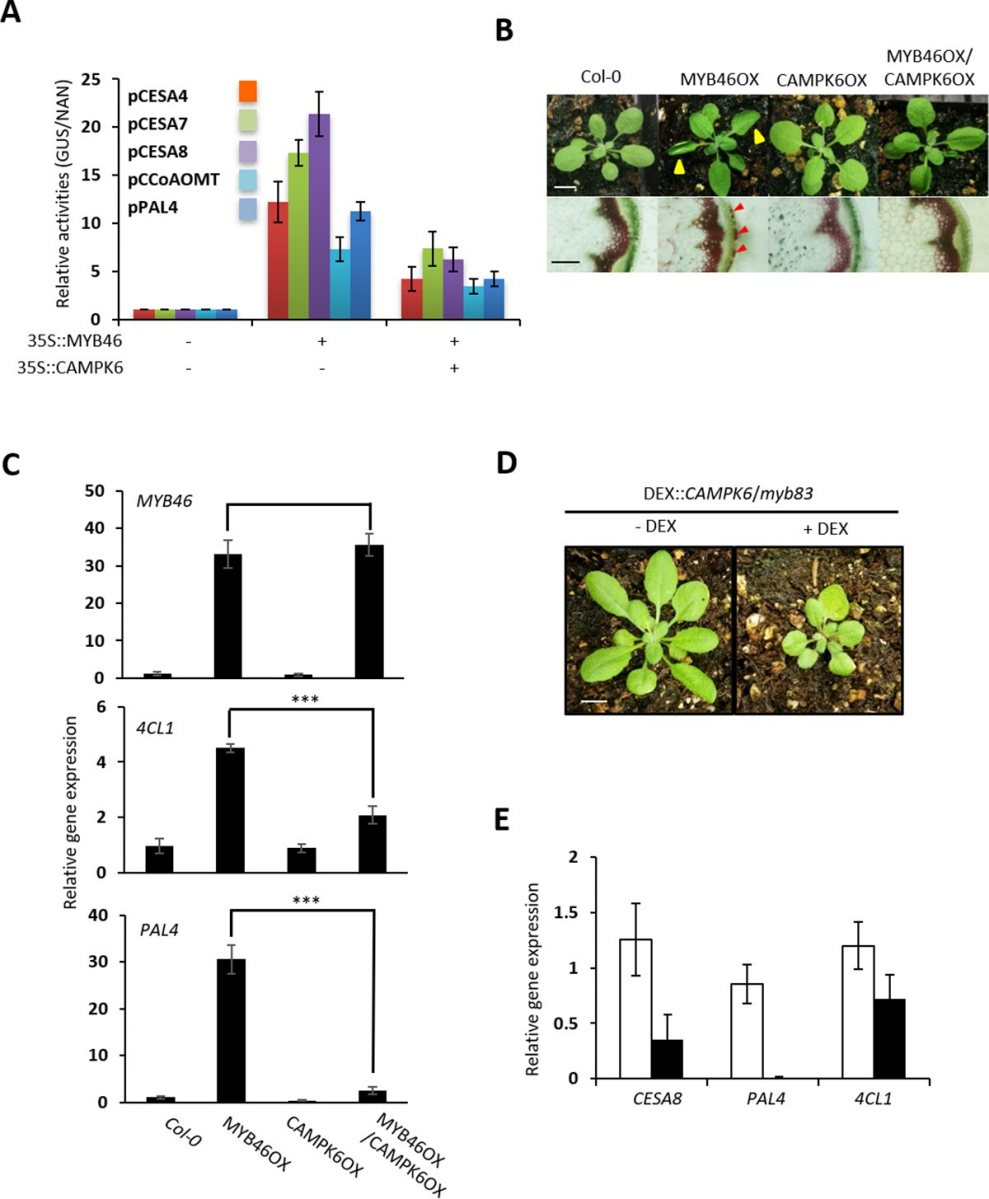
MPK6 negatively regulates MYB46 function *in planta*. (A) Promoter activities of MYB46 target genes were decreased by *CAMPK6* co-expression. The promoter::GUS fusion constructs were expressed in AMPs with *MYB46* or *MYB46* and *CAMPK6*. NAN was used as expression control. (B) Phenotypic analysis of 3-week-old Col-0, MYB46OX, CAMPK6OX or MYB46OX/CAMPK6OX (upper panel; scale bar, 1 cm). Phloroglucinol-HCl (i.e., lignin) staining of stems of 8-weeks-old plants (lower panel; scale bar, 100 μm). Red arrowheads indicate ectopic lignifications. (C) Expression of *MYB46* and two direct targets (*4CL1* and *PAL4*) of MYB46. Relative expression of genes was measured by Real-Time qPCR analysis using 3-week-old wild-type (Col-0) or transgenic *Arabidopsis* plants overexpressing *MYB46* (MYB46OX*)*, CAMPK6 (CAMPK6OX*)* or both (MYB46OX/CAMPK6OX). *** indicates statistically significant (p ≤ 0.01, t-test, n = 3). (D) DEX inducible expression of *CAMPK6* in the background of *myb83* mutant plants. Scale bar, 1 cm. (E) Expression of MYB46 target genes in *DEX*::*CAMPK6*/*myb83* plants with (black bars) or without (white bars) DEX treatment.

We expected that this MPK6-mediated degradation would negatively affect the function of MYB46 *in planta*. To test this hypothesis, we performed a transient transcription activation assay (TAA) using promoter sequences of known direct targets of MYB46 (e.g., *CESA4*, *CESA7*, *CESA8*, *CCoAOMT*, and *PAL4* to drive a *GUS* reporter gene as described previously [6]. When co-expressed with *35S* promoter-driven *MYB46* (*35S*::*MYB46*), GUS activities of the reporter constructs were dramatically increased (Fig 3A). However, the increased GUS activities were suppressed when co-expressed with *CAMPK6* (*35S*::*CAMPK6*), suggesting CAMPK6 negatively regulates MYB46 activity.

Upward leaf curling and ectopic secondary wall thickening, a typical phenotypes of constitutive *MYB46* overexpression plants [5], were clearly reproduced in the MYB46OX plants (Fig 3B). However, these phenotypes were reverted back to wild type in the MYB46OX/CAMPK6OX plants (Fig 3B). Upward leaf curling (Fig 3B, upper panel) and ectopic lignin staining (Fig 3B, lower panel) in MYB46OX were disappeared in the MYB46OX/CAMPK6OX plants (Fig 3B).

As expected, expression of two direct target genes of MYB46, *4CL1* and *PAL4*, was increased in the MYB46OX plants compared to Col-0 plants (Fig 3C). However, even though *MYB46* gene expression in MYB46OX/CAMPK6OX plants was similar to that in MYB46OX plants, expression of *4CL1* and *PAL4* were reduced significantly in the MYB46OX/CAMPK6OX plants, further suggesting CAMPK6 negatively regulates MYB46 activity (Fig 3C).

To further confirm the MPK6-mediated negative regulation of MYB46 function, we created transgenic *Arabidopsis* plants expressing dexamethasone (DEX)-inducible *CAMPK6* in the *myb83* mutant background (*DEX*::*CAMPK6*/*myb83*); MYB83 is a paralog of MYB46 in *Arabidopsis* [20]. With DEX treatment, the *DEX*::*CAMPK6*/*myb83* plants exhibited stunted growth (Fig 3D), which is a typical phenotype of *myb46/myb83* double knockout mutants [20]. This observation suggests that CAMPK6 regulates MYB46 protein function, negatively. Accordingly, the expressions of MYB46 target genes were decreased with DEX treatment in the *DEX*::*CAMPK6*/*myb83* plants (Fig 3E). Taken together, we suggest that MPK6-mediated phosphorylation negatively regulates MYB46 protein activity *in planta*.

### Functional significance of MPK6 phosphorylation target sites of MYB46

**Fig 4:**
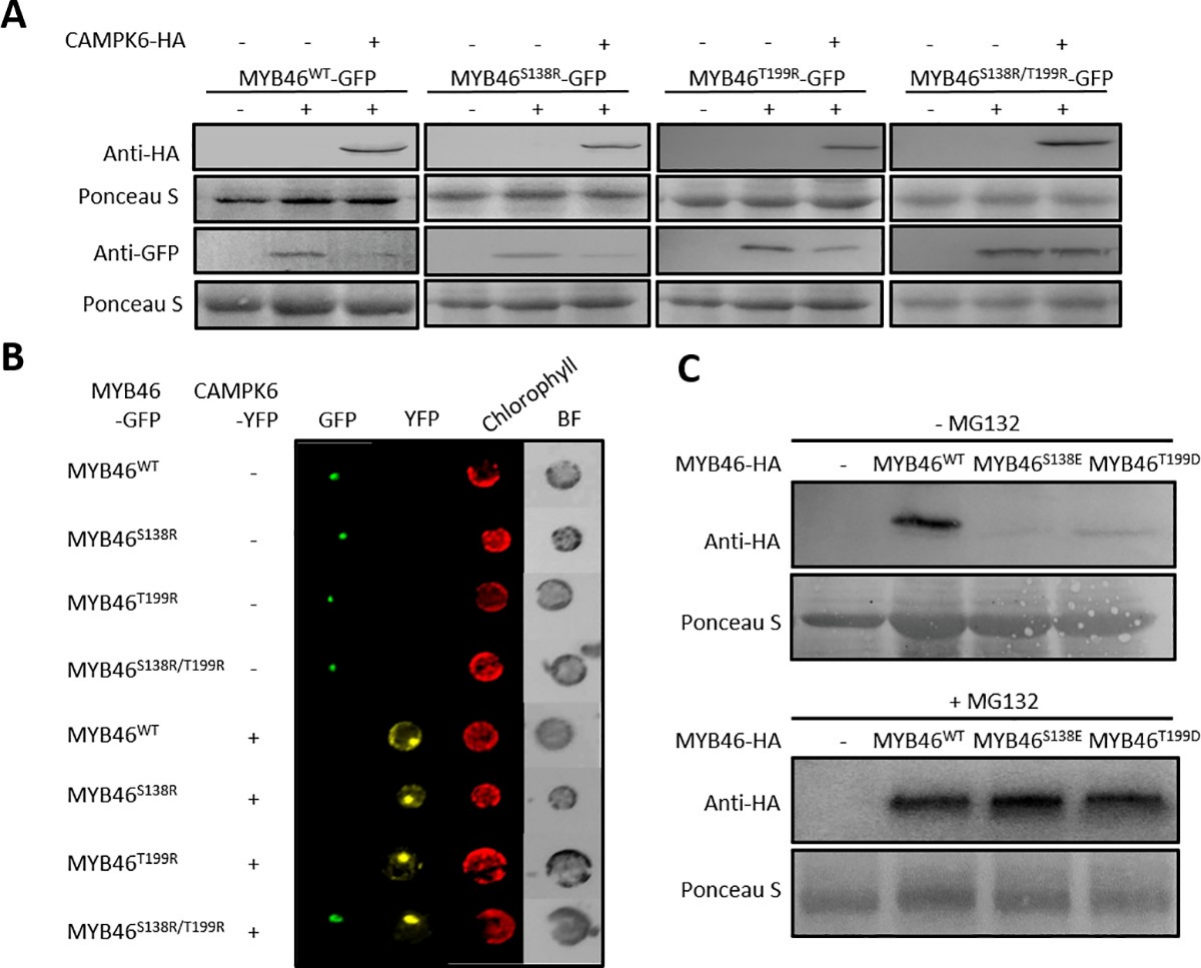
Functional significance of MPK6 phosphorylation target sites of MYB46. (A) Protein blot analysis of MYB46 and its non-phosphorable mutants. The MYB46-GFP and its non-phosphorable mutants (MYB46^S138R^-GFP, MYB46^T199R^-GFP, or MYB46^S138R/T199R^-GFP) were expressed with or without CAMPK6-HA in AMPs and incubated for 10-hr. Protein blot analysis was carried out with anti-GFP antibody for MYB46 and anti-HA antibody for CAMPK6. (B) MYB46-GFP and its non-phosphorable mutants were expressed with CAMPK6-YFP. After 10-hr incubation, image was taken by fluorescence microscopy. (C) From AMPs, protein blot analysis of MYB46-HA and its phospho-mimics (MYB46^S138E^ or MYB46^T199D^) with or without proteasome inhibitor MG132. The protein blot analysis was carried out with anti-HA antibody.

MYB46 has two putative MPK phosphorylation sites, S138 and T199 (Fig 1A). To test the functional significance of the phosphorylation sites, we point-mutated MYB46 to make a single non-phosphorable form (either S138R or T199R) or double non-phosphorable form (S138R/T199R) [21, 22]. We first assessed whether those mutant proteins were subject to MPK6-mediated degradation by using protein blot analysis. Each of the single mutant proteins, MYB46^S138R^ or MYB46^T199R^, were degraded when co-expressed with CAMPK6 in AMPs, while the double mutant protein MYB46^S138/T199R^ was not (Fig 4A), suggesting that a single phosphorylation is sufficient for MYB46 protein degradation by CAMPK6. This was further confirmed by co-expressing CAMPK6-YFP and MYB46-GFP fusion proteins in AMPs. The GFP signal was detected when wild-type MYB46 (*35S*::*MYB46* ^*wt*^-*GFP*) or mutant MYB46 (*35S*::*MYB46*^*S138R*^-*GFP*, *35S*::*MYB46*^*T199R*^-*GFP*, or *35S*::*MYB46*^*S138R/T199R*^-*GFP*) was expressed without CAMPK6 (*35S*::*CAMPK6*-*YFP*) co-expression. However, the GFP signal disappeared when *MYB46*-*GFP*, *MYB46*^*S138R*^-*GFP*, or *MYB46*^*T199R*^-*GFP* was co-expressed with *CAMPK6*-*YFP*, respectively (Fig 4B). Consistent with the protein blot analysis result (Fig 4A), the GFP signal was still detected from MYB46^S138R/T199R^-GFP (*35S*::*MYB46*^*S138R/T199R*^-*GFP*) even in the presence of CAMPK6-YFP (Fig 4B). Taken together, we suggest that phosphorylation at either one of the target sites is sufficient for MPK6-mediated degradation of MYB46 protein.

The functional significance of the two phosphorylation sites was further tested by introducing phosphomimic mutations into the two sites (either MYB46^S138E^ or MYB46^T199D^). Phosphomimic mutation at either one of the target sites resulted in degradation of MYB46 protein (Figs 4C and S2), confirming that phosphorylation of the two target sites leads to degradation of MYB46. Accordingly, the expression of MYB46 target genes was significantly reduced in the phosphomimic mutant line (MYB46^S138E^) when compared to MYB46OX line, despite that the expression of *MYB46* itself was not different (S2B Fig). However, the degradation was not observed in the presence of proteasome inhibitor MG132 (Fig 4C). In addition, substitution of lysine with arginine at a putative ubiquitination site of MYB46 (K156R) prevented degradation of the mutant MYB46 protein (S3 Fig), further confirming that this phosphorylation-dependent degradation of MYB46 is through the ubiquitin-mediated proteasome pathway.

### Phosphorylation sites of MYB46 is important for *in planta* MYB46 activity

**Fig 5:**
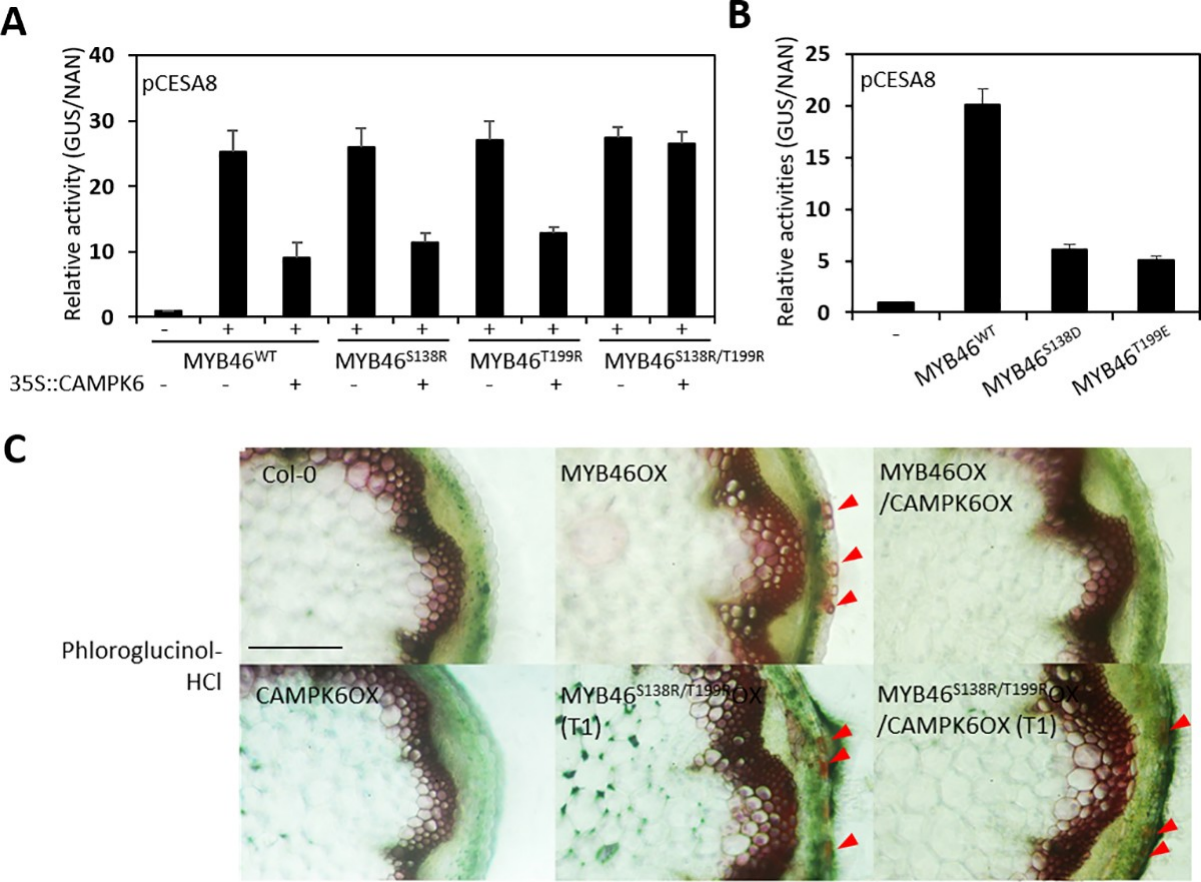
Phosphorylation sites of MYB46 is important for *in planta* MYB46 activity. (A) pCESA8::*GUS* was expressed with MYB46 or its non-phosphorable mutants (MYB46^S138R^, MYB46^T199R^, or MYB46^S138R/T199R^) with or without CAMPK6 in AMPs. After 6-hr incubation, the cells were harvested for measurement of GUS activity. NAN was used as expression control. (B) pCESA8::*GUS* was expressed in AMPs with MYB46 or its phospho-mimic mutants (MYB46^S138E^ or MYB46^T199D^). After 6-hr incubation, GUS activity was measured and NAN was used as expression control. (C) Lignin staining of stem sections. Phloroglucinol-HCl staining was performed on the rosette-level stem sections of 8-week-old Col-0 plants, transgenic plants overexpressing MYB46 (MYB46OX), MYB46 and CAMPK6 (MYB46OX/CAMPK6OX), non-phosphorable mutant (MYB46^S138R/ T199R^OX, T1), or MYB46^S138R/T199R^ and CAMPK6 (MYB46^S138R/T199R^OX/CAMPK6OX, T1). Red arrowheads indicate ectopic lignifications. Scale bar, 100 μm.

To test whether the site specific phosphorylation of MYB46 affects *in planta* MYB46 activity, first, we performed a TAA by co-expressing MYB46 mutant constructs with the *GUS* reporter gene driven by the *CESA8* promoter (*pCESA8*::*GUS*), with or without *CAMPK6* in AMPs. As expected, GUS activities of *pCESA8* were increased in wild-type (MYB46^wt^) and all the non-phosphorable MYB46 mutant constructs (MYB46^S138R^, MYB46^T199R^, and MYB46^S138R/T199R^). However, the increased GUS activities were compromised, significantly when co-expressed with *CAMPK6* (*35S*::*CAMPK6*) except in the double MYB46 mutants (MYB46^S138R/T199R^), suggesting that CAMPK6-mediated site specific phosphorylation of MYB46 affects MYB46 activity (Fig 5A). In addition, phosphomimic mutation at either one of the two phosphorylation sites resulted in a significant reduction in MYB46 activity (Fig 5B).

To further confirm these findings, we produced transgenic *Arabidopsis* plants overexpressing *MYB46*^*wt*^ or *MYB46*^*S138R*/*T199R*^ with or without *CAMPK6*. The transgenic plants overexpressing wild-type *MYB46* (*35S*::*MYB46*^*wt*^) or with double non-phosphorable mutations (*35S*::*MYB46*^*S138R*/*T199R*^) showed ectopic lignification in the epidermal cells (a typical phenotype of constitutive *MYB46* overexpression) without *CAMPK6* co-expression. However, that ectopic lignification disappeared in the *35S*::*MYB46*^*wt*^ transgenic plants co-expressing *CAMPK6* (i.e., MYB46OX/CAMPK6OX), while the double non-phosphorable mutants co-expressing *CAMPK6* (i.e., MYB46 ^S138R/T199R^OX/CAMPK6OX) was showed the ectopic lignification (Figs 5C and S4). These observations further suggest that MPK6 negatively regulates MYB46 function *in planta* through site specific phosphorylation-dependent degradation of MYB46.

### MYB83, a paralog of MYB46, is not regulated by MPK6

**Fig 6:**
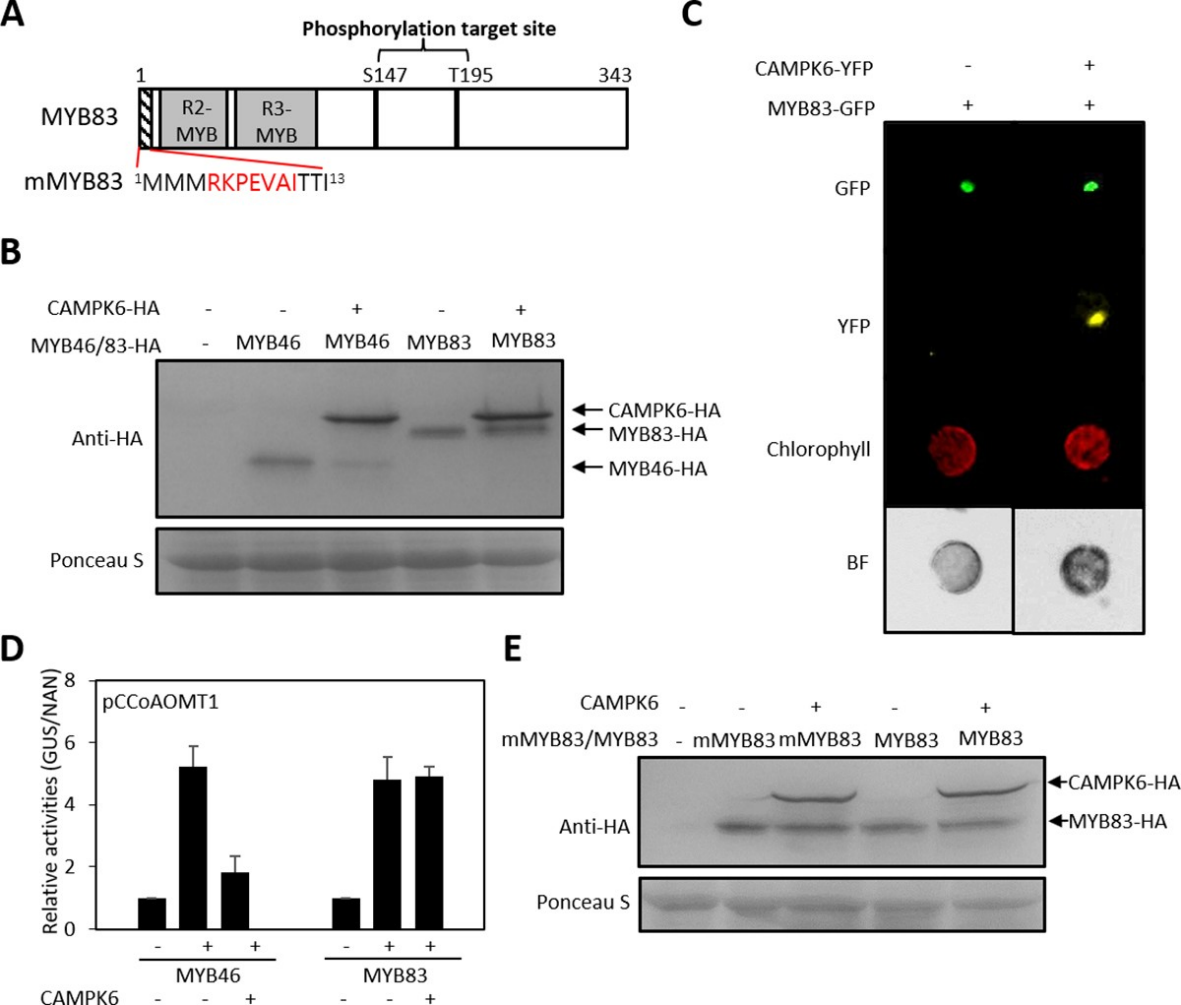
MYB83, a paralog of MYB46, is not regulated by MPK6. (A) Schematic drawing of phosphorylation target sites in MYB83 predicted by Eukaryotic Linear Motif (http://elm.eu.org/). MPK docking domain was not identified in MYB83 but the same MPK docking domain of MYB46 was introduced to produce mMYB83 as shown. (B) Protein blot analysis of MYB83. MYB46-HA or MYB83-HA was transfected with CAMPK6 in designated combinations in AMPs. After 10-hr incubation, protein blot analysis was carried out with anti-HA antibody. (C) MYB83-GFP signal. MYB83-GFP expressed in AMPs with or without CAMPK6-YFP. Image was taken after incubation for 10-hr by fluorescence microscopy. (D) *CCoAOMT* promoter activities. *CCoAOMT*::*GUS* was transfected to AMPs with MYB46, MYB83 and CAMPK6 in designated combinations. After 6-hr incubation, the cells were harvested and GUS activities were measured. NAN was used as expression control. (E) Stability of the MPK docking motif introduced MYB83 mutant protein (mMYB83) was not affected by CAMPK6. Protein blot analysis of both MYB83 and mMYB83 was done with CAMPK6 co-expression.

Based on the ELM prediction, MYB83 has two putative phosphorylation target sites, S147 and S195 but no MPK docking domain, which is unlike to MYB46 (Fig 6A). Thus, we hypothesized that MYB83 is not a substrate for MPK6. We tested this hypothesis by investigating whether MYB83 is degraded by MPK6. To do this, we carried out protein blot analysis of MYB83-HA fusion proteins expressed in AMPs with or without CAMPK6. MYB46-HA was included as a positive control. MYB83 protein level was not changed regardless of CAMPK6 co-expression while MYB46 protein was degraded in the presence of CAMPK6 (Fig 6B), suggesting that MYB83 is not a substrate for MPK6. This observation was confirmed by expressing MYB83-GFP fusion construct (*35S*::*MYB83*-*GFP*) in AMPs with or without CAMPK6-YFP fusion construct (*35S*::*CAMPK6*-*YFP*). GFP signal was detected in the MYB83 regardless of CAMPK6 co-expression (Fig 6C).

Since MYB83 protein stability is not affected by MPK6, we expected its function may not be regulated by CAMPK6. To test this hypothesis, we used a *GUS* reporter gene driven by the promoter of *CCoAOMT1*, a direct target of both MYB46 and MYB83 [20] in TAA using AMPs (Fig 6D). GUS expression was increased by either *MYB46* or *MYB83* expression without *CAMPK6* co-expression but the MYB46-induced GUS activity was reduced when *CAMPK6* was co-expressed. However, this decrease was not observed with MYB83 even in the presence of CAMPK6, suggesting that MYB83 is not regulated by MPK6.

To see if the absence of N-terminal MPK docking domain in MYB83 is the reason why MPK6 does not regulate MYB83 activity, we introduced the same MPK docking domain of MYB46 to the N-terminal of MYB83 (mMYB83, Fig 6A). However, the stability of the resulting mMYB83 was not affected by CAMPK6 (Fig 6E). Thus, these results further support the conclusion that MYB83 is not regulated by MPK6.

## Discussion

The R2R3-MYB transcription factor MYB46 functions as a master switch for secondary cell wall biosynthesis, which is critical for growth of vascular plants [4, 5, 20]. Talyor-Teeples et al [9] reported that salt stress increased *MYB46* gene expression but did not alter the expressions of its direct target genes (e.g., lignin biosynthetic genes) in *Arabidopsis* roots, suggesting an existence of post-transcriptional or translational regulatory mechanism of MYB46 activity. In this study, we demonstrated that MYB46 activity is post-translationally regulated by direct binding of MPK6 through various biochemical and genetic experiments.

The mitogen-activated protein kinase (MPK) cascade is a highly conserved signal transduction module involved in transducing extracellular signals (e.g., positional and environmental information) to the nucleus for appropriate biochemical and physiological cellular responses. *In silico* analysis showed that MYB46 has a putative MPK-docking domain and two phosphorylation target sites, suggesting that MPK signaling may be involved in the regulation of MYB46 function. Firstly, we found that MPK6 phosphorylates MYB46 protein with a direct interaction by employing an Y2H assay, BiFC experiments, co-IP and immunocomplex kinase assay (Fig 1). Next, we showed that MYB46 protein stability is negatively regulated by the site-specific phosphorylation of MYB46 by CAMPK6 through ubiquitin mediated proteasome pathway (Figs 2 and 4). Finally, we demonstrated that MPK6-mediated MYB46 phosphorylation regulates *in planta* secondary wall forming function of MYB46 (Figs 3 and 5). In addition, we provide experimental evidences that MYB83, a paralog of MYB46, is not regulated by MPK6 (Fig 6).

Initially, we focused on both MPK3 and MPK6 because they are closely related [23, 24] and post-translationally activated by salt stress [11, 16, 25]. In order to study their ability to target MYB46 as substrate, we used constitutively-active form of MPK3 (i.e., CAMPK3) and MPK6 (i.e., CAMPK6) because they do not require their upstream kinases for activation [26]. Both CAMPK3 and CAMPK6 have been shown to phosphorylate conventional MPK phosphorylation sites and interact with upstream kinases as well as their substrates [27]. Furthermore, the mutations that led to the constitutively active form do not affect substrate specificity [27]. Indeed, CAMPK6 showed stronger phosphorylation activity than MPK6 (S5 Fig). Transcriptional activity and protein stability of MYB46 were significantly changed with *CAMPK6* co-expression but not with *CAMPK3* co-expression in AMPs (S1 Fig), suggesting that MYB46 may not be a substrate of MPK3. While both MPK3 and MPK6 are commonly activated by many environmental stress factors, their substrate specificity appears to be diverse. A kinase assay on a protein microarray carrying 1,690 Arabidopsis proteins identified 48 potential substrates of MPK3 and 39 potential substrates of MPK6, respectively, and only 26 of which were common substrates for both kinases [28].

In mammalian system, phosphorylation and ubiquitin-mediated proteasome pathway are prominent means of post-translational regulation of the activity of transcription factors [29, 30]. Many plant transcription factors (TFs) are also known to have their activity regulated by phosphorylation [22, 31–36]. MPK-TF interactions lead to either negative or positive regulation of the activity of the TFs. We found that MYB46 protein stability is negatively regulated by MPK6 *via* ubiquitin-mediated proteasome pathway (Fig 2), resulting in reduction of its transcriptional activity (Fig 3).

Little is known about the post-translational regulation of MYB46, a master switch of secondary wall biosynthesis, and the interplay between environmental signaling and secondary wall biosynthesis. Our MPK6-MYB46 regulatory module provides a novel insight into a mechanism by which plants incorporate environmental signals into differential regulation of secondary wall synthesis. The advantages of this post-translational regulation are the speed and specificity of plant response to environmental changes. Thus, this MPK6-MYB46 regulatory module may afford the affected plant a rapid and efficient means to cope with those unexpected and quickly changing abiotic stresses which occur in nature.

## Materials and methods

### Plant growth conditions

For phenotypic analysis, *A*. *thaliana* plants were grown in soil under a photoperiod of 16-hr light/8-hr dark at 24°C. For protoplast generation, plants were grown in soil for 23 to 25 days under a photoperiod of 13-hr light/11-hr dark at 23°C. The humidity was adjusted to 50%.

### Generation of *Arabidopsis* mesophyll protoplasts (AMP)s and transient activation assay (TAA)

Protoplast generation and TAA were performed as described previously [6, 22]. For the effector constructs, full-length gene constructs were ligated between the CaMV *35S* promoter and the *nopaline synthase terminator* after removing *GUS* from the pTrGUS vector. Reporter constructs were used as previously described [5]. The primers used for PCR amplification of full-length genes and point-mutants are listed in S1 Table. For protein blot analysis and detection of GFP signal, genes were cloned in *HA* and *GFP* tagged HBT vector [18]. Plasmid DNA was prepared using a Plasmid Plus Maxi kit (QIAGEN, Valencia, CA, USA), and used for transfection as shown in S2 Table. For internal control for GUS activity normalization, 6 μg of NAN plasmid [37] was added. Then, 17 μl of plasmid mixture (34 μg) and 200 μl of protoplasts were transferred to 2 ml microcentrifuge tubes following the procedure described by [37]. β-Glucuronidase and NAN enzyme assays were performed as described by [38]. NAN and GUS activities were measured using MUN (Sigma-Aldrich Co.) and MUG (Sigma-Aldrich Co.) as substrates, respectively, against MU standards on a SpectraMax M2 Microplate Readers (excitation: 365 nm, emission: 455). The ratio of GUS and NAN activities is represented as relative GUS/NAN units. Three biological replicates were used in the experiments.

### Protein blot analysis

The cells were lysed using sodium dodecyl sulphate (SDS) protein sample buffer and heated at 95°C for 5 min after transfection with designated plasmid combinations. The protein blot analysis was performed as described previously [18]. Anti-MYB46 antibody was purchased from Abmart (http://www.ab-mart.com/).

### Yeast two hybrid assay

Interaction of MYB46 with CAMPK6 was confirmed by His auxotrophic growth of yeast. Yeast cells (AH109), transformed with the plasmid pairs indicated in Fig 1B, were cultured on synthetic dropout (SD) medium for nutritional selection. After 4 days, colony was transferred to SD-Leu-Trp-His + 1 mM 3AT, an SD medium without Leu, Trp, His with 1 mM 3AT, and spot assay was carried out.

### BiFC Assay

*MYB46*-*EYFP*, *EYFP*-*MYB46*, *CAMPK6*-*EYFP*, and *EYFP*-*CAMPK6* constructs using pSAT4-DEST-nEYFP-C1, pSAT5-DEST-cEYFP-C1, pSAT4(A)-DEST-nEYFP-N1 and pSAT5(A)-DEST-cEYFP-N1 were transfected to AMPs with designated combinations in Fig 1D (for PCR primer and vector information, see S1 and S2 Tables). After 10-hr incubation, the cells were observed and imaged with a fluorescence microscope (339 Leica DRAMA2 Fluorescent microscope).

### Immunocomplex kinase assay

*MYB46-GFP* construct and *CAMPK6-HA* construct were transfected to AMPs and incubated for 10-hr. After incubation, immunocomplex kinase assay was performed as described previously [38]. Briefly, after transfection, the cells were disrupted and the supernatant was used in agarose-immunoprecipitation. Immunocomplexed agarose was washed and phosphorylated using the phosphorylation buffer (20 mM Tris-HC1 [pH 7.5], 40 mM MgCl_2_, 5mM EDTA, 1 mM DTT, 0.1 mM ATP, 0.25 mg ml^-1^ and 50 μM [γ-^32^P] ATP) for 30 min at room temperature. The samples were separated with SDS-PAGE and phosphorylation was detected with phosphor-image analyzer (FLA-7000, Fujifilm, Japan) after gel dry.

### Co-immunoprecipitation assay

After 1-hr co-transfection of GFP conjugated to MYB46 and HA conjugated to CAMPK6 in AMPs, the cells were treated with 1 μL of 5 mM of MG132 and additionally incubated 10-hr at room temperature. The immunoprecipitation step was followed by immunocomplex kinase assay method and protein blot analysis was carried out with anti-HA antibody and anti-GFP antibody.

### Phloroglucinol-HCl staining

*Arabidopsis* root or rosette-level stem sections were stained with 2% phloroglucinol-HCl for 3 min and imaged by microscope (Diaphot Inverted ELWD0.3, Nikon).

### qRT-PCR analysis

Total RNA was extracted from leaves of 2- or 3-week-old *Arabidopsis* plants using RNeasy Plant Mini Kit (Qiagen). For cDNA synthesis, SuperScript™ II Reverse Transcriptase (Invitrogen) was used. qPCR was carried out with specific primers on 7500 Real-Time PCR System (Applied Biosystems) using comparative Ct method with Fast SYBR™ Green Master Mix (Applied Biosystems). Means of log-transformed data were compared by t-test using PROC TTEST in SAS v9.4 (SAS Institute, Chicago, IL, USA), and statistical significance was determined at 3 levels (α = 0.1, α = 0.05, α = 0.01; n = 3).

### DEX treatment

DEX-inducible *CAMPK6*/*myb83* plants were grown in soil for 2.5 weeks, then sprayed with 5 μM DEX + 0.015% Silwet L-77 + 0.05% ethanol. After 24-hr, leaves were harvested for qRT-PCR. Plants were sprayed three additional times at 3 day intervals, and photographed 1 week after the final treatment.

## Supporting information

S1 Fig MYB46 transcriptional activity and its protein stability are not regulated by CAMPK3, a constitutively activated MPK3. (A) *CESA8* promoter activities. *pCESA8*::*GUS* construct was transfected to AMPs with *MYB46*, *CAMPK3* and *CAMPK6* in designated combinations. After 6-hr incubation the cells were harvested and GUS activities were measured. NAN was used as expression control. (B) MYB46-GFP signal. *MYB46*-*GFP* construct was expressed in AMPs with or without *CAMPK3*. CAMPK6 was used as positive control. Image was taken after incubation for 10-hr by fluorescence microscopy. (C) Protein blot analysis of MYB46. *MYB46-HA* was transfected with or without *CAMPK3 or CAMPK6* in designated combinations in AMPs. After 10-hr incubation protein blot analysis was carried out with anti-HA antibody.
(TIF)

S2 Fig Phosphomimic mutation at either one of the target sites resulted in degradation of MYB46 protein. (A) Protein blot analysis from 3-weeks-old T_1_ transgenic plants overexpressing MYB46 (MYB46OX) or its phospho-mimics (MYB46^S138E^). The proteins were detected with anti-MYB46 antibodies. (B) Gene expression analysis of MYB46 and its direct target genes in 3-weeks-old T_1_ transgenic plants overexpressing MYB46 (MYB46OX) or its phospho-mimics (MYB46^S138E^). The qRT-PCR was carried out with gene specific primers.
(TIF)

S3 Fig Mutation of putative ubiquitination site increases the stability of phosphormimic MYB46 mutant protein. Ubiquitination site was predicted from UbPred (http://www.ubpred.org/) and Lys156 was predicted as a putative ubiquitination site. Lys156 to Arg mutations were done in two phosphormimic mutant MYB46 proteins, and protein blot analysis was carried out.
(TIF)

S4 Fig Ectopic lignification by overexpression of *MYB46*^*S138R/ T199R*^ is not reduced by CAMPK6. Stem anatomical observation was done using 8-week-old designated plants. Rosette-level stems were sectioned by hand and stained with phloroglucinol-HCl and then imaged by microscope. Col-0 and MYB46OX (A); T_1_ lines of MYB46^S138R/ T199R^OX T_1_ plants (B); MYB46^S138R/ T199R^OX/CAMPK6OX T_1_ plants (C). Red arrowheads indicate ectopic lignifications. Scale bar, 100 μm.
(TIF)

S5 Fig CAMPK6, a constitutively-active MPK6, negatively regulates MYB46 activity. (A) *In vitro* kinase assay of MPK6 and CAMPK6. *MPK6-HA* and *CAMPK6-HA* were expressed in AMPs. Immunoprecipitation was carried out using anti-HA antibody and followed by kinase assay. Myelin Basic Protein (MBP) was used as a substrate. (B) MYB46 dependently induced *CESA8* promoter activity is reduced by CAMPK6 co-expression. After 6-hr of incubation, the cells were harvested for GUS activity measurement. NAN was used as expression control.
(TIF)

S1 Table Sequence of primers used in this study. (PDF)

S2 Table Promoter and effector construct combinations in PEG-transfection. (PDF)
