# Supplementary figures and images for: Mitogen-activated protein kinase 6 negatively regulates secondary wall biosynthesis by modulating MYB46 protein stability in Arabidopsis thaliana
Source: PLoS Genet. 2021 Apr 7;17(4):e1009510. doi: 10.1371/journal.pgen.1009510 (PMC8055014; doi:10.1371/journal.pgen.1009510)

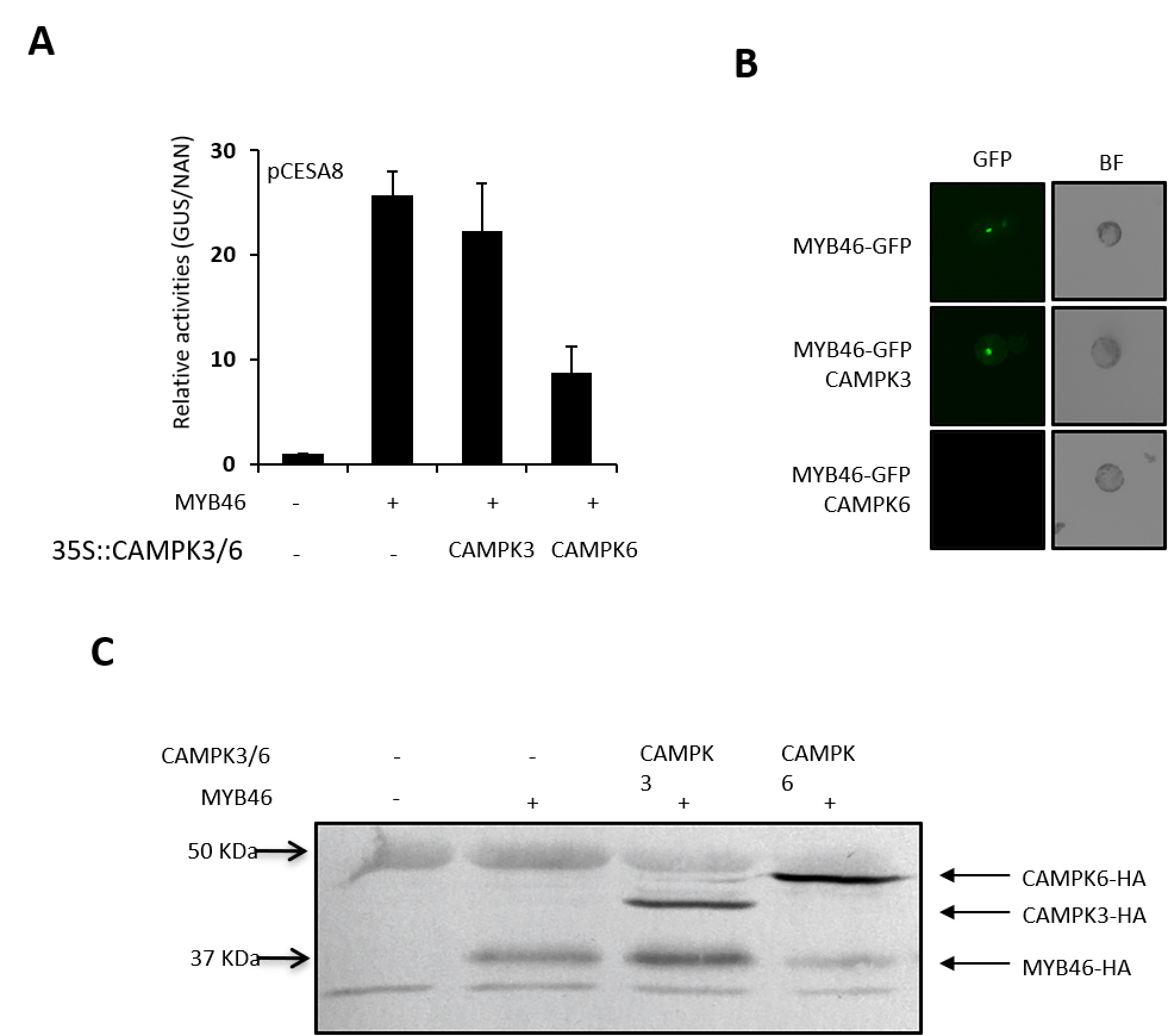

Supplement: S1 Fig — (A) CESA8 promoter activities. pCESA8::GUS construct was transfected to AMPs with MYB46, CAMPK3 and CAMPK6 in designated combinations. After 6-hr incubation the cells were harvested and GUS activities were measured. NAN was used as expression control. (B) MYB46-GFP signal. MYB46-GFP construct was expressed in AMPs with or without CAMPK3. CAMPK6 was used as positive control. Image was taken after incubation for 10-hr by fluorescence microscopy. (C) Protein blot analysis of MYB46. MYB46-HA was transfected with or without CAMPK3 or CAMPK6 in designated combinations in AMPs. After 10-hr incubation protein blot analysis was carried out with anti-HA antibody. (TIF) [file pgen.1009510.s001.tif]

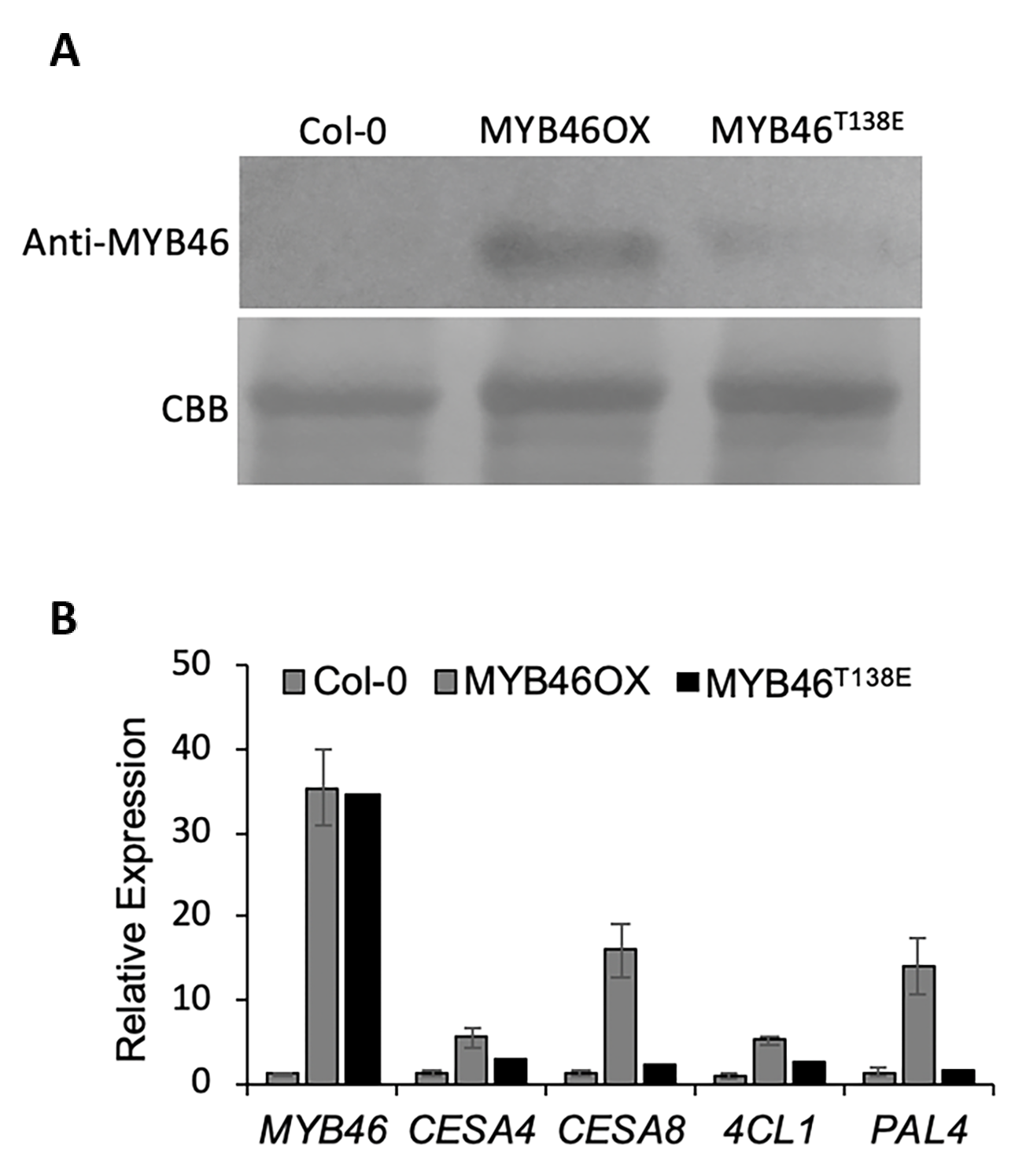

Supplement: S2 Fig — (A) Protein blot analysis from 3-weeks-old T1 transgenic plants overexpressing MYB46 (MYB46OX) or its phospho-mimics (MYB46S138E). The proteins were detected with anti-MYB46 antibodies. (B) Gene expression analysis of MYB46 and its direct target genes in 3-weeks-old T1 transgenic plants overexpressing MYB46 (MYB46OX) or its phospho-mimics (MYB46S138E). The qRT-PCR was carried out with gene specific primers. (TIF) [file pgen.1009510.s002.tif]

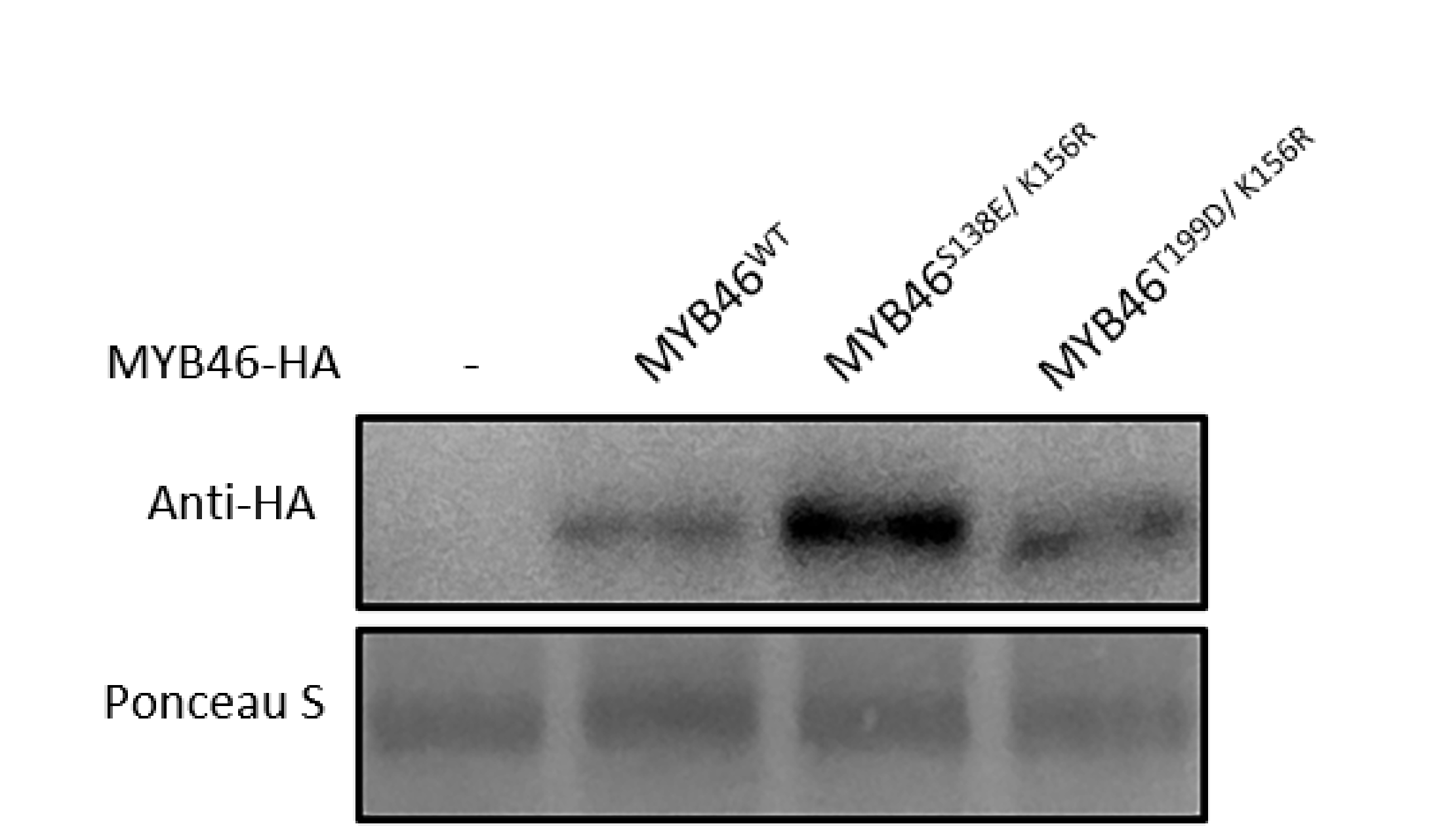

Supplement: S3 Fig — Ubiquitination site was predicted from UbPred (http://www.ubpred.org/) and Lys156 was predicted as a putative ubiquitination site. Lys156 to Arg mutations were done in two phosphormimic mutant MYB46 proteins, and protein blot analysis was carried out. (TIF) [file pgen.1009510.s003.tif]

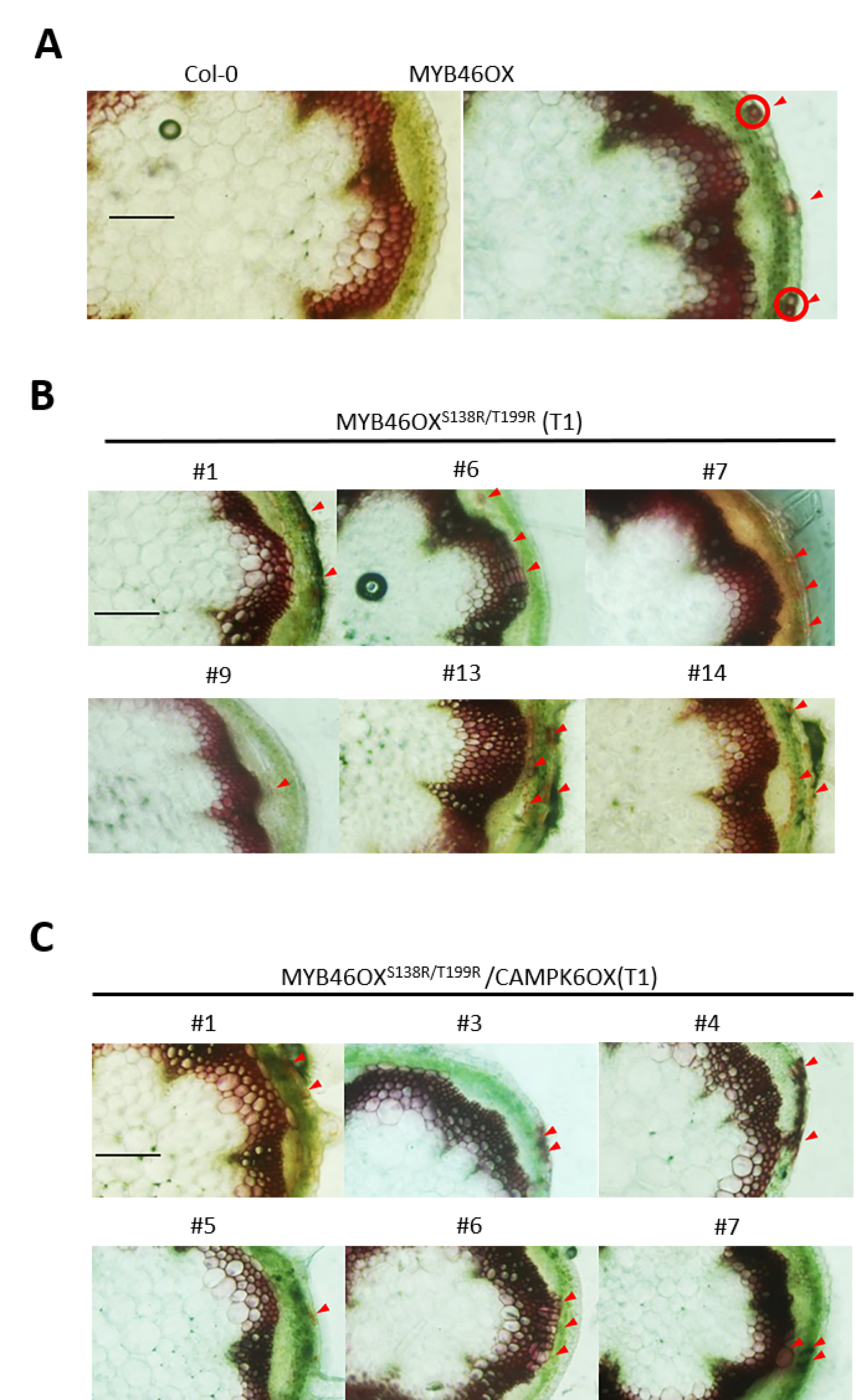

Supplement: S4 Fig — Stem anatomical observation was done using 8-week-old designated plants. Rosette-level stems were sectioned by hand and stained with phloroglucinol-HCl and then imaged by microscope. Col-0 and MYB46OX (A); T1 lines of MYB46S138R/ T199ROX T1 plants (B); MYB46S138R/ T199ROX/CAMPK6OX T1 plants (C). Red arrowheads indicate ectopic lignifications. Scale bar, 100 μm. (TIF) [file pgen.1009510.s004.tif]

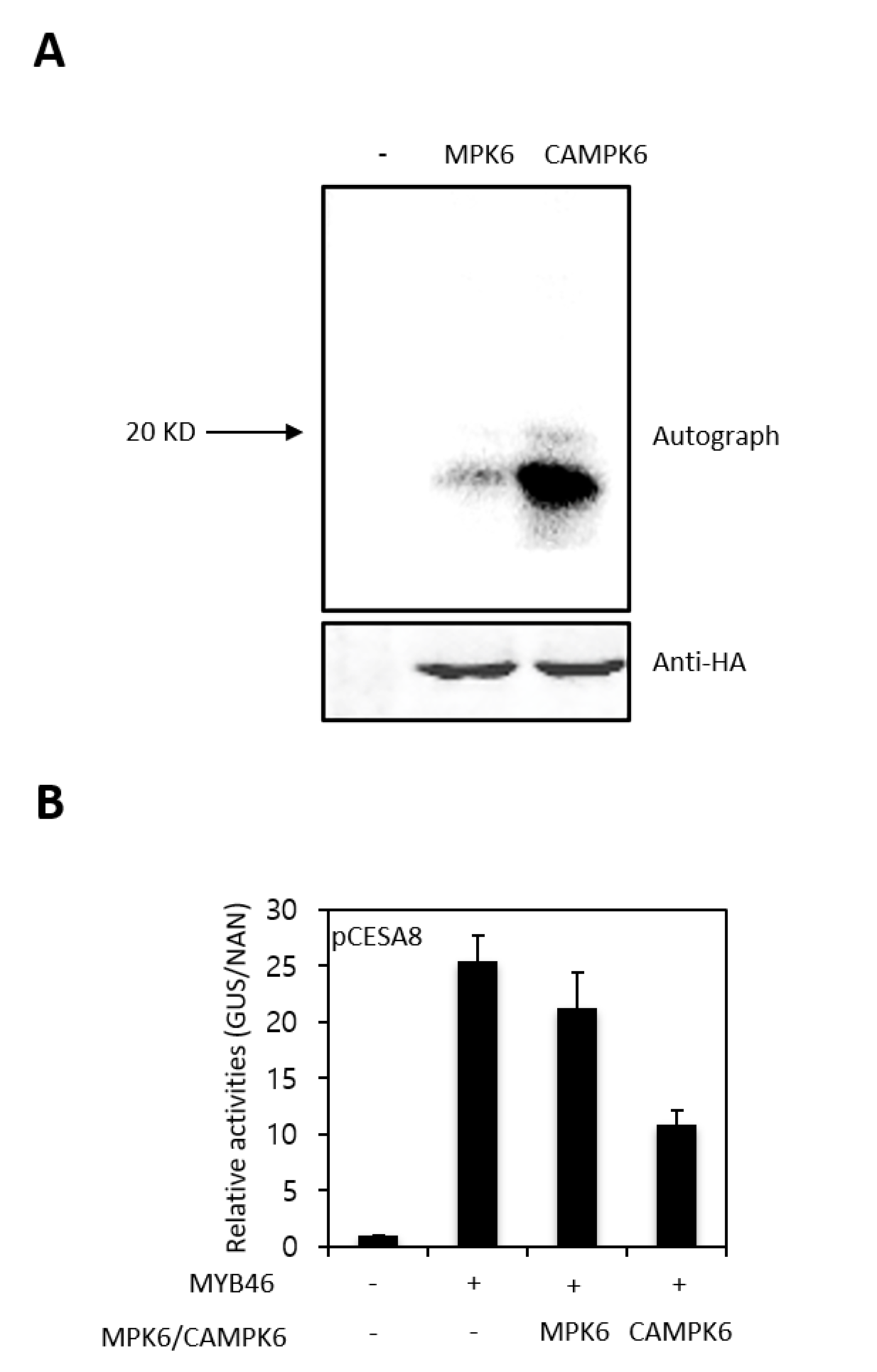

Supplement: S5 Fig — (A) In vitro kinase assay of MPK6 and CAMPK6. MPK6-HA and CAMPK6-HA were expressed in AMPs. Immunoprecipitation was carried out using anti-HA antibody and followed by kinase assay. Myelin Basic Protein (MBP) was used as a substrate. (B) MYB46 dependently induced CESA8 promoter activity is reduced by CAMPK6 co-expression. After 6-hr of incubation, the cells were harvested for GUS activity measurement. NAN was used as expression control. (TIF) [file pgen.1009510.s005.tif]
