# Supplementary material for: Mitogen-activated protein kinase 6 negatively regulates secondary wall biosynthesis by modulating MYB46 protein stability in Arabidopsis thaliana
Source: PLoS Genet. 2021 Apr 7;17(4):e1009510. doi: 10.1371/journal.pgen.1009510 (PMC8055014; doi:10.1371/journal.pgen.1009510)
Supplement: S1 Table — (PDF) [file pgen.1009510.s006.pdf]

Supplemental Table S1. Sequence of primers used in this study.

Purpose	Name	Sequence (5' to 3')
Cloning	MYB46-F	CATGCCATGGCAAGGAAGCCAGAGGTAGC
	MYB46-R	GAAGGCCTTATGCTTTGTTTGAAGTTGA
	MPK6-F	CGGGATCCATGGACGGTGGTTCAGGTCA
	MPK6-R	GAAGGCCTTTGCTGATATTCTGGATTGA
	MYB46-EYFP_M46_F	AAAAAGCAGGCTATGAGGAAGCCAGAGGTAGCCAT
	MYB46-EYFP_M46_R	AGAAAGCTGGGTTTATGCTTTGTTTGAAGTTGAAGT
	EYFP-MYB46_M46_F	AAAAAGCAGGCTCCATGAGGAAGCCAGAGGTAGCCAT
	EYFP-MYB46_M46_R	AGAAAGCTGGGTTCATATGCTTTGTTTGAAGTTGA
	CAMPK6-EYFP_F	AAAAAGCAGGCTATGGACGGTG GTTCAGGTCA
	CAMPK6-EYFP_R	AGAAAGCTGGGTTTTGCTGATATTCTGGATTGAAAGCA
	EYFP-CAMPK6_F	AAAAAGCAGGCTCCATGGACGGTG GTTCAGGTCA
	EYFP-CAMPK6_R	AGAAAGCTGGGTTTGCTGATATTCTGGATTGAAAGCATGA
	MYB83-F	CATGCCATGGCCATGATGATGAGGAAACCGGA
	MYB83-R	CCCCCGGGATCGACTTGGAAATCAAGGAA
Mutagenesis	CAMPK6-F	TGAGAGTGGTTTCATGACTGCATATGTTGT
	CAMPK6-R	ACAACATATGCAGTCATGAAACCACTCTCA
	$MYB46^{S138R}$-F	ACTCATCCTCAAGACCCAACACAGCAAGCG
	$MYB46^{S138R}$-R	CGCTTGCTGTGTTGGGTCTTGAGGATGAGT
	$MYB46^{T199R}$-F	GCAATGACGATTTTAGACCTTATGTAGATG
	$MYB46^{T199R}$-R	CATCTACATAAGGTCTAAAATCGTCATTGC
	$MYB46^{S138E}$-F	ACTCATCCTCAGAACCCAACACAGCAAGCG
	$MYB46^{S138E}$-R	CGCTTGCTGTGTTGGGTTCTGAGGATGAGT
	$MYB46^{T199D}$-F	GCAATGACGATTTTGACCCTTATGTAGATG
	$MYB46^{T199D}$-R	CATCTACATAAGGGTCAAAATCGTCATTGC
qRT-PCR	4CL1-F	AGGTTCCTTTGCAAAACCTAACGA
	4CL1-R	CGATAAGAGTGGTGAAATCTGGTGC
	PAL4-F	GGCGGTGCACTTCAAAATGA
	PAL4-R	GAGAATCTCGAAGCGTATACCGGA
	PP2A-F	TAACGTGGCCAAAATGATGC
	PP2A-R	GTTCTCCACAACCGCTTGGT
	MYB46-q-F	ATCGGACATCTTCTTTAGCCTTTTCTT
	MYB46-q-R	CTCAAGCGTGGCGCTTTCT
