# Supplementary material for: Mitogen-activated protein kinase 6 negatively regulates secondary wall biosynthesis by modulating MYB46 protein stability in Arabidopsis thaliana
Source: PLoS Genet. 2021 Apr 7;17(4):e1009510. doi: 10.1371/journal.pgen.1009510 (PMC8055014; doi:10.1371/journal.pgen.1009510)
Supplement: S2 Table — (PDF) [file pgen.1009510.s007.pdf]

Supplemental Table S2. Promoter and effector construct combinations in PEG-transfection.

Figure	pTr-GUS	pTr-NAN	Effectors	Control DNA	Total (µg)
Fig. 1D	0	0	20 µg of *MYB46*, 20 µg of *CAMPK6*	20 µg	40 µg
Fig. 2A	0	0	20 µg of *MYB46*, 20 µg of *CAMPK6*	20 µg	40 µg
Fig. 2B	0	0	30 µg of *MYB46*, 30 µg of *MPK6*/CAMPK6	30 µg	60 µg
Fig. 2C	0	0	30 µg of *MYB46*, 30 µg of *CAMPK6*	30 µg	60 µg
Fig. 3A	0	0	30 µg of *MYB46*s, 30 µg of *CAMPK6*	30 µg	60 µg
Fig. 3B	0	0	30 µg of *MYB46*s, 30 µg of *CAMPK6*	30 µg	60 µg
Fig. 3C	0	0	30 µg of *MYB46*s	30 µg	30 µg
Fig. 4A	6 µg of promoter	6 µg	14 µg of *MYB46*, 14 µg of CA*MPK6*	0-28 µg	40 µg
Fig. 5A	6 µg of promoter	6 µg	14 µg of *MYB46s*, 14 µg of CA*MPK6*	0-28 µg	40 µg
Fig. 5B	6 µg of promoter	6 µg	14 µg of *MYB46s*	14 µg	40 µg
Fig. 6B	0	0	30 µg of *MYB46/83*, 30 µg of *CAMPK6*	30 µg	60 µg
Fig. 6C	0	0	30 µg of *MYB83*, 30 µg of *CAMPK6*	30 µg	60 µg
Fig. 6D	6 µg of promoter	0	14 µg of *MYB46 or MYB83* 14 µg of CA*MPK6*	0-28 µg	40 µg
Fig. 6E	0	0	30 µg of *MYB83* or *mMYB83*, 30 µg of *CAMPK6*	30 µg	60 µg
